# Suicidal ideation in male UK military personnel who sustained a physical combat injury in Afghanistan and the mediating role of leaving service: The ADVANCE cohort study

**DOI:** 10.1177/00207640241264195

**Published:** 2024-07-31

**Authors:** Daniel Dyball, Charlotte Williamson, Alexander N Bennett, Susie Schofield, Christopher J Boos, Anthony MJ Bull, Paul Cullinan, Nicola T Fear

**Affiliations:** 1 King’s Centre for Military Health Research, King’s College London, UK; 2 Academic Department of Military Rehabilitation, Defence Medical Rehabilitation Centre, Stanford Hall Estate, Nottinghamshire, UK; 3 National Heart and Lung Institute, Faculty of Medicine, Imperial College London, UK; 4 Faculty of Health & Social Sciences, Bournemouth University, UK; 5 Centre for Injury Studies, Department of Bioengineering, Imperial College London, UK; 6 Academic Department of Military Mental Health, King’s College London, UK

**Keywords:** Afghanistan, military personnel, suicidal ideation, wounds and injuries, ADVANCE cohort

## Abstract

**Background/aims:**

Suicidal Ideation (SI) is a risk factor for suicide, a leading cause of death amongst young men globally. In this study we assess whether sustaining a serious physical combat injury is associated with SI and whether leaving service mediates this association.

**Methods:**

We analysed data from male UK Armed Forces personnel who sustained a combat injury in Afghanistan and a frequency-matched comparison group who did not sustain such an injury (the ADVANCE cohort). SI was measured from the Patient Health Questionnaire-9 item ‘thoughts that you would be better off dead or of hurting yourself in some way’.

**Results:**

Approximately, 11.9% (*n* = 61) of the uninjured group, 15.3% (*n* = 83) of the overall injured group, 8.5% (*n* = 13) of an Amputation injury (AI) subgroup and 17.6% (*n* = 70) of a Non-Amputation Injury (NAI) subgroup reported SI in the past 2 weeks. The NAI subgroup reported greater likelihood of SI (Relative Risk Ratio (RR) = 1.44, 95% confidence interval (CI) [1.04, 2.00]) compared to the comparison group, whereas the overall injured group (RR = 1.23, 95% CI [0.90, 1.68]) and AI subgroup (RR = 0.65, 95% CI [0.36, 1.18]) did not. Leaving service fully mediated the association between sustaining a NAI and SI (natural direct effect RR = 1.08, 95% CI [0.69, 1.69]).

**Conclusions:**

UK military personnel with NAI reported significantly higher rates of SI compared to demographically similar uninjured personnel, while those who sustained AIs reported no significant difference. Leaving service was associated with greater rates of SI for both injured and uninjured personnel and fully mediated the association between sustaining a NAI and SI.

## Introduction

### Suicidal ideation

Suicide is one of the leading causes of mortality amongst young men globally (Pitman et al., 2012). Suicidal Ideation (SI), which includes thoughts, ideas or ruminations about the possibility of ending one’s life, may precede suicide attempts and is a risk factor for suicidal behaviours (e.g. self-harm and making suicide plans; Harmer et al., 2020; May et al., 2015; Park et al., 2017; World Health Organisation, 1992). Whilst active SI (e.g. a desire to kill oneself) is thought to convey greater risk for suicidal behaviour, even passive SI, such as feelings that one would be better off dead, carries with it a considerable risk (Baca-Garcia et al., 2011; Liu et al., 2020; May et al., 2015). Mental illness is a significant risk factor for engaging in suicide-related behaviours, which is at least partially driven through increases in SI (Jakupcak et al., 2009; Ribeiro et al., 2018).

### Suicide risk in the UK military

UK veterans have previously been identified as having more than double the prevalence of intentional self-harm (self-harm or suicide attempt; 10.5%) compared to still-serving personnel (4.2%; Pinder et al., 2012). An investigation into a cohort of regular UK Armed Forces who left military service between 1996 and 2018 found that overall suicide rates among veterans were slightly lower when compared to the UK general population (standardised mortality ratio = 94 (95% confidence interval (CI) [88, 99]; Rodway et al., 2023), a finding that was also reported amongst Scottish veterans (Bergman et al., 2022). However, these studies found an increased risk among UK veterans under the age of 25 years old (age-specific mortality ratio = 160, 95% CI [136, 187] in those aged 20–24 years) and Scottish early service leaver veterans (personnel who leave the Armed Forces with less than 3 years of service) when compared to non-veterans (hazard ratio = 1.25, 95% CI [1.05, 1.41]).

During the UK military deployments to Afghanistan (2002–2014, Operation HERRICK) a considerable number of Armed Forces personnel survived severe physical combat injuries (McGuire et al., 2019). In the US and Canada, sustaining a combat injury has been found to be associated with suicide attempts and SI (Boulos & Fikretoglu, 2018; Walker et al., 2023). An investigation into UK male military personnel (median age at injury 25 years, Inter Quartile Range (IQR) = 22–29 years) from the ADVANCE cohort, a cohort of personnel who sustained a serious physical combat injury compared to a frequency matched comparison group who did not sustain a serious physical combat injury, found that combat injury was associated with greater rates of depression, anxiety and PTSD, though type of injury was an important modifier (Dyball et al., 2022). Those who sustained amputation injuries reported similar rates of mental illness compared to the comparison group, whereas those who sustained non-amputation injuries reported significantly higher rates. This suggests that non-amputation injuries may be associated with higher rates of reported SI compared to those who sustained amputation injuries and demographically similar uninjured personnel.

### Aims

In this analysis, we present the rates of SI in a group of UK Armed Forces personnel who deployed to Afghanistan and sustained a physical combat injury and compare them to the rates in an uninjured, frequency-matched comparison group. We also compare injured subgroups (amputation injury and non-amputation injury) to the comparison group and investigate the mediating effect of leaving service on the association between sustaining a physical combat injury and SI. We hypothesised that those who sustained physical non-amputation combat injuries, but not those who sustained amputation-related injuries, will report greater rates of SI compared to the comparison group. We also hypothesised that leaving service will mediate the association between sustaining an injury and SI.

## Methods

### Participants

The ADVANCE cohort is composed of a group of UK military personnel who sustained a serious physical combat injury in Afghanistan and a frequency matched uninjured comparison group. A total of 579 injured male personnel were recruited into the baseline data assessment and 566 uninjured who were frequency-matched on sex, age, rank, role on deployment, regiment and deployment era. The sample was provided by the UK Ministry of Defence: Defence Statistics department. Eligibility criteria for the injured group included having sustained a physical combat injury during a deployment to Afghanistan; having an aeromedical evacuation due to the injury which resulted in admission to a UK hospital; and no history of cardiovascular, liver or renal disease before injury. Eligibility criteria for the uninjured group included having deployed to Afghanistan and sustaining no serious physical combat injuries; and no history of cardiovascular, liver or renal disease prior to deployment. The ADVANCE protocol provides full details of the study design and sampling (Bennett et al., 2020). This analysis uses data from the baseline assessment of the ADVANCE cohort, which is ambidirectional in nature. The response rate of eligible participants in the overall injured group was 59.6% and 62.6% for the amputation injury subgroup (Dyball et al., 2022).

### Procedure

Participants attended the Defence Medical Rehabilitation Centre (DMRC) Headley Court (2015–2018) or Stanford Hall (2018–2020) and took part in a comprehensive set of health investigations, including a research nurse-led clinical interview as well as completion of a confidential self-report questionnaire.

### Materials

#### Outcome variable

##### Suicidal ideation

SI was measured using responses to the Patient Health Questionnaire-9 (PHQ9) item ‘Over the last 2 weeks, how often have you been bothered by thoughts that you would be better off dead or of hurting yourself in some way?’ (Kroenke et al., 2001). Answers ranged from 0 ‘not at all’, 1 ‘several days’ 2 ‘more than half the days’ and 3 ‘nearly every day’. Scores were binary coded to indicate ‘no SI in the past 2 weeks’ (score 0) and ‘any SI in the past 2 weeks’ (score 1–3).

#### Independent variables

##### Combat injury

Combat injury was established from electronic medical records, information provided by the Ministry of Defence: Defence Statistics department and supplemented by participants self-reports during the research nurse-led clinical interview. Amputation injuries were defined as major limb amputation (e.g. transhumeral and transtibial). Isolated partial amputation (e.g. digit, toe and partial foot) without major limb amputation were not included in the amputation injury group unless accompanied by major limb amputation.

New Injury Severity Scores (NISS), which range from a score of 1 to 75, were extracted from the UK Joint Theatre Trauma Registry. The NISS was treated as a continuous variable and also categorised around the median observed NISS score (NISS ⩾ 13).

#### Confounders

##### Age

Age at assessment in years was used.

##### Combat role at sampling

Combat role was established during the research nurse-led clinical interview. Roles were coded as ‘combat support/combat service support/other role’ for example, Royal Engineers or medics, and ‘combat role’, for example, infantry.

##### Socioeconomic status

Rank at sampling was used as a proxy for socioeconomic status; junior non-commissioned officer rank/other rank (NATO OR2–OR4), senior non-commissioned officer rank (NATO OR5–OR9) and commissioned officer rank (NATO OF1–OF6; Yoong et al., 1999).

##### Data analysis

Data analysis was conducted using the statistical software package STATA MP version 18.0. Sampling weights and response weights were calculated and applied to demographic tables as weighted percentages and presented alongside raw cell counts (Dyball et al., 2022). The primary exposure explored in this analysis is sustaining a physical combat injury and type of injury (e.g. non-amputation injury and amputation injury).

Generalised linear models with log link were used to assess un-mediated direct effects of sustaining a physical combat injury on SI and results are presented as Relative Risk Ratios (RR). Counterfactual mediation was undertaken using the ‘paramed’ function of STATA with a loglinear function for the dependent variable (SI) and a logistic function for the mediator (leaving service). This approach was chosen to allow for a common binary mediator and binary outcome, and also assessment (and allowance) of mediator-outcome interactions (Emsley & Liu, 2013).

**Figure 1.**
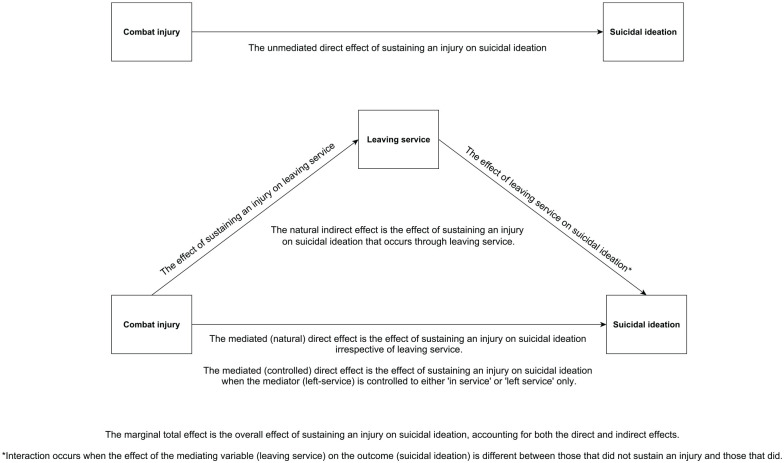
Directed Acyclic Graph explaining unmediated effects, mediated effects and interactions.

The hypothetical directed acyclic graph can be found in Figure 1 along with descriptions of the unmediated direct effect, natural direct effect, controlled direct effect, marginal total effect and interaction terms. Coefficients are presented for unmediated direct effects and RRs are presented for mediated effects. Standard errors were calculated using 1,000 bootstrap replications for generalised linear models and mediation analyses. Bias-corrected confidence intervals are presented.

The amputation injury subgroup was excluded from mediation models due to low rates of SI and only a small number of still serving personnel (*n* = 17/161), many of whom were in the process of transitioning out of service. Confounders were included based on a-priori chosen variables: age at assessment (Hilderink et al., 2015; Moradi et al., 2021), combat role (as a proxy for combat experiences; Bryan et al., 2015; Stevelink et al., 2018) and rank at sampling (as a proxy for socioeconomic status; Madigan & Daly, 2023).

##### Missing data

Five participants (<1%) had missing data for the PHQ9 item on SI. These participants were excluded from analysis, alongside one participant from the comparison group who was excluded due to sustaining a severe injury outside military service.

##### Ethics

The authors assert that all procedures contributing to this work comply with the ethical standards of the relevant national and institutional committees on human experimentation and with the Helsinki Declaration of 1975, as revised in 2008. The Ministry of Defence Research Ethics Committee approved the study (ref: MODREC protocol No:357/PPE/12). All participants provided written informed consent.

## Results

A total of 1,139/1,145 participants (99.5% of ADVANCE cohort) were included in this analysis; 563 in the comparison group, 576 in the injured group, 160 in the amputation injury subgroup and 416 in the non-amputation injury subgroup. Median NISS was higher in the amputation injury subgroup (NISS = 24.5 (Interquartile range (IQR) = 17, 34)) compared to the non-amputation injury subgroup (NISS 9 (IQR = 4, 17). The bodily areas in which the injured group sustained injuries and differences between the amputation injury subgroup and non-amputation injury subgroup have been reported elsewhere (Dyball et al., 2022; Supplemental Appendix). Overall, 144 participants reported SI (13.8%, 95% CI [11.8, 16.0]). 99 participants (9.2%) reported experiencing SI for several days in the last 2 weeks, 32 participants (3.2%) reported experiencing SI on more than half the days in the last 2 weeks and 13 participants (1.4%) reported experiencing SI nearly every day in the last 2 weeks. Sociodemographic data are reported in Table 1 stratified by combat injury groups. Rates of SI stratified by physical combat injury and veteran status are presented in Table 2. The still-serving uninjured group reported the lowest rates of SI (8.3%), while the veteran uninjured group reported the greatest rates of SI (28.9%) across all examined groups. Rates of SI stratified by physical combat injury, rank at sampling, tertiled age ranges and combat role, are available in Supplemental Materials 1.

**Table 1.** Sociodemographic and military characteristics, overall and by physical combat injury status.

	Comparison group (*n* = 563)	Injured group (*n* = 576)	Amputation injury subgroup (*n* = 160)	Non-amputation injury subgroup (*n* = 416)
Sociodemographics				
Age in years at time of injury/deployment of interest, median (IQR)	26 (23, 29)	25 (22, 29)	25 (22, 28)	25 (22, 29)
Age in years at assessment, median (IQR)	34 (30, 37)	33 (30, 37)	32.5 (30, 36)	33 (30, 38)
Ethnicity (*n*)				
White	90.5% (511)	90.2% (521)	91.5% (147)	89.7% (374)
All other ethnic groups	9.5% (52)	9.8% (55)	8.5% (13)	10.3% (42)
Rank at sampled deployment (*n*)				
Junior non-commissioned officer/other rank	66.5% (338)	76.4% (411)	84% (127)	73.8% (284)
Senior non-commissioned officer	24.6% (146)	17.3% (106)	11% (20)	19.4% (86)
Officer	8.9% (79)	6.3% (59)	5% (13)	6.8% (46)
Combat role (*n*)				
Combat support/combat service support/other role	19.4% (114)	15.1% (89)	15.1% (26)	15.1% (63)
Combat role	80.6% (448)	84.9% (486)	84.9% (134)	84.9% (352)
Serving status (*n*)				
	82.3% (465)	26.9% (157)	10.4% (17)	32.6% (140)
*Veteran*	17.7% (98)	73.1% (419)	89.6% (143)	67.4% (276)
New Injury Severity Score (NISS)				
**1–12 (*<*50^th^ percentile)**	**–**	**56.9% (311)**	**18.1% (28)**	**70.4% (283)**
**13–75 (*≥*50^th^ percentile)**	**–**	**43.1% (265)**	**81.9% (132)**	**29.6% (133)**

*Note*. Weighted percentages are presented alongside unweighted cell counts. IQR = interquartile range.

**Table 2.** Rates of suicidal ideation, stratified by combat injury and serving status.

Suicidal Ideation in the last two weeks (Patient Health Questionnaire 9-item 9)	Uninjured (*n* = 563)			Injured (*n* = 576)			Amputation injury subgroup (*n* = 160)			Non-amputation injury subgroup (*n* = 416)		
	% (95% confidence interval) (*n*)			% (95% confidence interval) (*n*)			% (95% confidence interval) (*n*)			% (95% confidence interval) (*n*)		
	Overall	In-service	Veteran	Overall	In-service	Veteran	Overall	In-service	Veteran	Overall	In-service	Veteran
Any suicidal ideation (several days-almost every day)	11.9% (9.3–15.1) (61)	8.3% (6.0–11.4) (36)	28.9% (20.2–39.4) (25)	15.3% (12.5–18.7) (83)	10.1% (6.2–16.1) (16)	17.2% (13.7–21.5) (67)	8.6% (5.0–14.4) (13)	NR~	8.1% (4.5–14.4) (11)	17.7% (14.1–21.9) (70)	9.9% (5.8–16.3) (14)	21.4% (16.8–27.0) (56)

*Note*. Weighted percentages and confidence intervals are presented alongside unweighted cell counts.

~ Some data suppressed to allow for medical confidentiality in line with Defence Statistics rounding policy (https://www.gov.uk/government/publications/defence-statistics-policies/ministry-of-defence-disclosure-control-and-rounding-policy).

**Figure 2.**
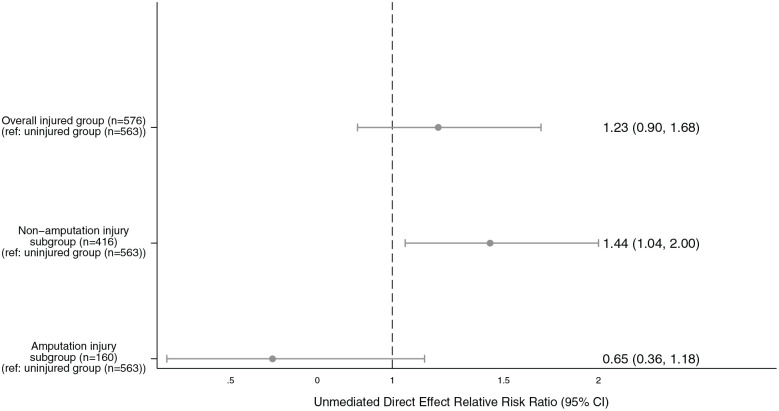
Unmediated direct effect of sustaining a physical combat injury on suicidal ideation using generalised linear models, controlling for age at assessment, combat role and socioeconomic status. *Bias-corrected confidence intervals bootstrapped with 1,000 replications*.

The unmediated direct effect estimates show that sustaining a physical combat injury was not associated with greater likelihood of reporting SI compared to the uninjured comparison group (RR = 1.23, 95% CI [0.90, 1.68]; Figure 2). The non-amputation injury subgroup reported a significantly greater likelihood of SI compared to the uninjured comparison group (RR = 1.44, 95% CI [1.04, 2.00]), whereas the likelihood of reporting SI for the amputation injury subgroup was not significantly different to the uninjured comparison group (RR = 0.65, 95% CI [0.36, 1.18]).

**Figure 3.**
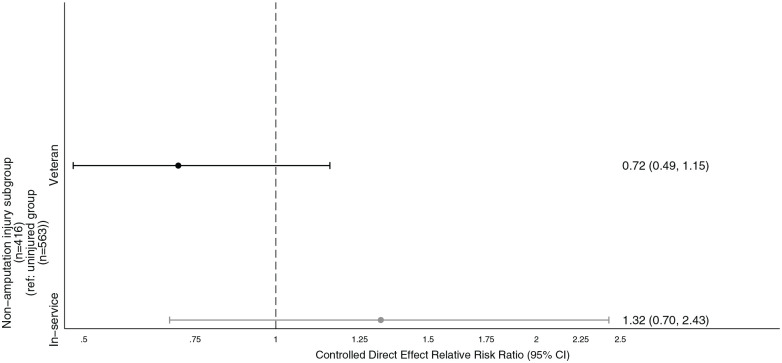
Controlled direct effect of sustaining a physical non-amputation combat injury on suicidal ideation in mediation model when effect is fixed to in-service versus veteran groups, controlling for age at assessment, combat role and socioeconomic status. *Bias-corrected confidence intervals bootstrapped with 1,000 replications*.

In the mediation analysis, the natural direct effect of sustaining a non-amputation injury on SI was not significantly different from demographically similar uninjured personnel (RR = 1.08, [0.69, 1.69]). Leaving service was associated with a greater likelihood of reporting SI (RR = 3.35, 95% CI [2.04, 5.50]). Sustaining a non-amputation injury had an indirect effect on SI through leaving service (natural indirect effect RR = 1.36, 95%CI [1.06, 1.88]). The controlled direct effect suggested that that rates of SI would not be significantly different between those that sustained non-amputation injuries and demographically similar uninjured personnel if all personnel had stayed in-service (RR = 1.32, 95% CI [0.70, 2.43]) or if all personnel had left service (RR = 0.72, 95% CI [0.49, 1.15]; Figure 3). The marginal total effect of sustaining a non-amputation injury on SI compared to demographically similar uninjured personnel was RR = 1.47, 95% CI [1.05, 2.11]. Overall, the mediation analysis suggests that leaving service fully mediates the association between sustaining a non-amputation injury and SI.

## Discussion

This paper investigates SI amongst a cohort of UK military personnel who sustained serious physical combat injuries and a demographically similar comparison group who did not sustain such combat injuries. We hypothesised that sustaining a non-amputation combat injury would be associated with greater likelihood of reporting SI compared to the comparison group, and that those who sustained amputation injuries would have a similar likelihood of reporting SI to the comparison group. Our results supported these hypotheses. However, evidence from the further analyses suggests that the increased risk observed in those who sustained non-amputation injuries is primarily due to the increased risk of leaving service, and mediation modelling indicates that both those who sustained non-amputation injuries and demographically similar uninjured personnel who leave service report similar rates of SI.

### Leaving service and suicidal ideation

Whilst the comparison group was matched to the injured group on a range of demographics, current serving status was not one of them. Several factors may be involved in the process of leaving service for UK military personnel who deployed to Afghanistan and did not sustain serious physical combat injuries. Non-combat related or minor physical injuries from deployments/training exercises may result in increased physical symptoms, reduced physical functioning or pain, which may limit one’s ability to perform military duties or reduce the appetite for individuals to continue serving in the military (Benedict et al., 2019; Larsson et al., 2009). Additionally, physical injuries are not the only type of injuries that might be sustained from deployments. Psychological injuries, such as anxiety, depression or PTSD, may inform one’s decision to leave the Armed Forces and subsequently influence SI (Bell et al., 2011; Brignone et al., 2017; Hoffmire et al., 2019). It is also possible that exposure to a greater number of combat experiences might be associated with a greater risk of discharge from the Armed Forces (Brooks Holliday & Pedersen, 2017).

There are several mechanisms by which leaving service might affect suicidal ideation. Social support is a protective factor for self-harm and suicide behaviours in serving and ex-serving military personnel (Williamson et al., 2024). However, it is noted that involvement in the military community (Vogt et al., 2022) or social participation outside of employment (Hatch et al., 2013) in veterans have both been shown to decrease over time after leaving military service. This indicates possible mechanisms by which social support might decrease in veterans, which in turn could increase the risk of suicidal ideation. Additionally, qualities around leaving service have been shown to be risk factors of suicide behaviours, such as non-voluntary discharge and leaving service at a younger age/with lower total length of service (Randles et al., 2023; Williamson et al., 2024). Difficulties transitioning from military to civilian life surrounding a loss of group identity and weak feelings of belonging to a community have also been associated with suicidal ideation in veterans (Thompson et al., 2019).

Considering the effect of leaving service is associated with greater likelihood of reporting SI for both those that sustain non-amputation injuries and the uninjured comparison group, it is especially interesting that veterans who sustained amputation injuries had considerably lower rates of SI (veterans in amputation injury subgroup 8.9%; veterans in non-amputation injury subgroup 21.4%; veterans in uninjured comparison group 28.9%). Previously we have reported that the incidence rate of anxiety, depression, PTSD and multimorbidity were lower in those that sustained amputation injuries compared to those that sustained non-amputation injuries (Dyball et al., 2022). It is possible that those with amputation injuries get additional support compared to those with non-amputation injuries or those who deploy in similar roles but leave service without a serious physical injury. This support may come in the form of access to charitable services, social perception of ‘heroic’ actions and a ‘hierarchy of wounding’ (Caddick et al., 2021; Roberts et al., 2021). Further research is required to understand the mechanisms by which those with amputation injuries are less likely to report SI, and whether this reduced risk is maintained as age-related conditions (e.g. age-related pain and deteriorations in mobility) become more prominent.

### Combat experiences

A previous meta-analysis has suggested that specific combat experiences, including seeing wounded or dead individuals, having killed in combat or witnessed someone killing in combat or witnessing ‘atrocities’ were associated with either SI or suicide attempts (Bryan et al., 2015). Combat experiences may be associated with SI amongst both injured and uninjured personnel in the ADVANCE cohort, and whilst we controlled for combat role as a proxy for combat experiences, no information was available for exposure to specific combat experiences.

### Clinical implications

The reduction of risk of suicide and associated behaviours such as SI and suicidal attempts are key clinical outcomes during rehabilitation and subsequent reintegration into civilian life in Armed Forces personnel. The current Armed Forces Suicide Prevention Strategy and Action Plan (UK Ministry of Defence, 2023) focusses on eight key areas, the first of which is understanding and identifying high risk groups within the Armed Forces (Defence, 2023). Findings from this analysis emphasise the importance of assessing both mental and physical health in those with serious physical combat injuries, with a suggestion that some serious injuries (specifically non-amputation) might carry increased risk. Further work is needed to understand why veterans who sustained non-amputation injuries and demographically similar uninjured veterans report greater SI than their still-serving counterparts. Continued observation of this cohort may help us understand whether changes in risk for SI will occur as military personnel with/without injury age, and age-related comorbidities become more prominent.

### Strengths/limitations

This analysis benefits from a large sample of injured personnel and a well-matched comparison group. However, there are some limitations. SI as measured by the PHQ has been found to have poor specificity and positive predictive value for measures of SI with intent and gold standard measures of SI (Na et al., 2018). SI is a heterogenous problem, representing sometimes only a fleeting desire for life to end, or more severe or intrusive thoughts regarding ending your life. Our study is unable to distinguish between individuals with passive or active SI, the latter of which may carry a higher risk of subsequent suicide attempt. It is of note however, that there is emerging evidence that passive SI or desire for death (Baca-Garcia et al., 2011) is not significantly different from active SI as a risk factor for many suicide-related behaviours (Liu et al., 2020). Several studies have reported evidence that higher scores on the PHQ9 item for SI are associated with greater likelihood of suicidal behaviour (Giddens & Sheehan, 2014; Simon et al., 2013), with one showing that a considerable number (23%) of those who scored only 1 (e.g. thoughts of being better off/dead for several days during several days over the past 2 weeks; the lowest score on the PHQ9 indicating SI) reported active SI at follow up (Walker et al., 2010). Whilst our mediation models are based on a temporally correct sequence of events (e.g. combat injury occurred before leaving service, which occurred before thoughts of SI in the past 2 weeks), it is possible that SI was present prior to leaving service. Interpretation of findings should take this into account. Un-measured mediator-outcome confounders cannot be ruled out. Finally and importantly, the uninjured veteran group in our cohort is relatively small, and not representative of all uninjured personnel who deployed to Afghanistan and left service. Rather, they are a subgroup of uninjured personnel who are representative of the qualities (e.g. rank, role on deployment, deployment era, regiment, age and sex) of personnel who sustained physical combat injuries.

## Conclusions

UK Armed Forces personnel who deployed to Afghanistan and sustained a non-amputation physical combat injury have a greater likelihood of reporting SI compared to demographically similar uninjured personnel. Personnel who sustained amputation injuries had no significant difference in likelihood of reporting SI compared to demographically similar uninjured personnel. Considerably greater risk of reporting SI appears to exist amongst veterans who sustained non-amputation injuries and demographically similar uninjured veterans compared to their still-serving counterparts.

## Supplemental Material

sj-docx-1-isp-10.1177_00207640241264195 – Supplemental material for Suicidal ideation in male UK military personnel who sustained a physical combat injury in Afghanistan and the mediating role of leaving service: The ADVANCE cohort study Supplemental material, sj-docx-1-isp-10.1177_00207640241264195 for Suicidal ideation in male UK military personnel who sustained a physical combat injury in Afghanistan and the mediating role of leaving service: The ADVANCE cohort study by Daniel Dyball, Charlotte Williamson, Alexander N Bennett, Susie Schofield, Christopher J Boos, Anthony MJ Bull, Paul Cullinan and Nicola T Fear in International Journal of Social Psychiatry
