# Supplementary material for: Suicidal ideation in male UK military personnel who sustained a physical combat injury in Afghanistan and the mediating role of leaving service: The ADVANCE cohort study
Source: Int J Soc Psychiatry. 2024 Jul 31;70(7):1279–88. doi: 10.1177/00207640241264195 (PMC11514323; doi:10.1177/00207640241264195)
Supplement: sj-docx-1-isp-10.1177_00207640241264195 – Supplemental material for Suicidal ideation in male UK military personnel who sustained a physical combat injury in Afghanistan and the mediating role of leaving service: The ADVANCE cohort study [file sj-docx-1-isp-10.1177_00207640241264195.docx]

Supplementary materials 1: Rates of suicidal ideation, stratified by combat injury and rank at sampling

| **Suicidal Ideation in the last two weeks**  (Patient Health Questionnaire 9-item 9) | **Uninjured (n=563)**  **% (95% Confidence Interval) (n)** | | | **Injured (n=576)**  **% (95% Confidence Interval) (n)** | | | **Amputation injury subgroup (n=160)**  **% (95% Confidence Interval) (n)** | | | **Non-amputation injury subgroup (n=416)**  **% (95% Confidence Interval) (n)** | | |
| --- | --- | --- | --- | --- | --- | --- | --- | --- | --- | --- | --- | --- |
|  | Junior NCO/Other rank | Senior NCO | Officer | Junior NCO/Other rank | Senior NCO | Officer | Junior NCO/Other rank | Senior NCO | Officer | Junior NCO/Other rank | Senior NCO | Officer |
| *Any suicidal ideation (Several days-almost every day)* | 15.0% (11.4, 19.4) (48) | 6.7% (3.6, 12.1) (10) | 3.7% (1.2, 11.3) (3) | 16.0% (12.6, 20.1) (63) | 16.1% (10.1, 24.6) (17) | 5.3% (1.6, 15.7) (3) | 8.1% (4.3, 14.7) (10) | 11.4% (2.5, 39.1) (2) | 9.4% (1.0, 52.2) (1) | 19.1% (14.8, 24.3) (53) | 17.0% (10.3, 26.6) (15) | 4.3% (1.0, 16.4) (2) |
| Weighted percentages and confidence intervals are presented alongside unweighted cell counts. | | | | | | | | | | | | |

| **Suicidal Ideation in the last two weeks**  (Patient Health Questionnaire 9-item 9) | **Uninjured (n=563)**  **% (95% Confidence Interval) (n)** | | | **Injured (n=576)**  **% (95% Confidence Interval) (n)** | | | **Amputation injury subgroup (n=160)**  **% (95% Confidence Interval) (n)** | | | **Non-amputation injury subgroup (n=416)**  **% (95% Confidence Interval) (n)** | | |
| --- | --- | --- | --- | --- | --- | --- | --- | --- | --- | --- | --- | --- |
|  | 23-31 years old | 32-36 years old | 37-59 years old | 23-31 years old | 32-36 years old | 37-59 years old | 23-31 years old | 32-36 years old | 37-59 years old | 23-31 years old | 32-36 years old | 37-59 years old |
| *Any suicidal ideation (Several days-almost every day)* | 14.0% (9.6, 19.8) (26) | 11.7% (7.7, 17.4) (21) | 8.7% (5.2, 14.3) (14) | 16.5% (11.8, 22.5) (32) | 14.6% (10.2, 20.6) (28) | 14.3% (9.6, 20.7) (23) | 10.1% (4.5, 21.1) (6) | 8.2% (3.3, 18.7) (5) | 5.3% (1.2, 20.3) (2) | 19.0% (13.1, 26.6) (26) | 17.0% (11.4, 24.6) (23) | 16.5% (10.9, 24.1) (21) |
| Weighted percentages and confidence intervals are presented alongside unweighted cell counts. | | | | | | | | | | | | |

Rates of suicidal ideation, stratified by combat injury and tertiled age ranges

| **Suicidal Ideation in the last two weeks**  (Patient Health Questionnaire 9-item 9) | **Uninjured (n=563) % (95% Confidence Interval) (n)** | | **Injured (n=576)**  **% (95% Confidence Interval) (n)** | | **Amputation injury subgroup (n=160)**  **% (95% Confidence Interval) (n)** | | **Non-amputation injury subgroup (n=416)**  **% (95% Confidence Interval) (n)** | |
| --- | --- | --- | --- | --- | --- | --- | --- | --- |
|  | Combat support/Combat service support | Combat | Combat support/Combat service support | Combat | Combat support/Combat service support | Combat | Combat support/Combat service support | Combat |
| *Any suicidal ideation (Several days-almost every day)* | 6.4% (3.0, 13.3) (7) | 13.3% (10.3, 17.0) (54) | 6.8% (3.2, 13.9) (7) | 16.9% (13.6, 20.7) (76) | 6.5% (1.4, 24.9) (2) | 8.9% (4.9, 15.6) (11) | 6.9% (2.8, 16.0) (5) | 19.6% (15.6, 24.4) (65) |
| Weighted percentages and confidence intervals are presented alongside unweighted cell counts. | | | | | | | | |

Rates of suicidal ideation, stratified by combat injury and combat role
